# Oxygen-Mediated Suppression of CD8+ T Cell Proliferation by Macrophages: Role of Pharmacological Inhibitors of HIF Degradation

**DOI:** 10.3389/fimmu.2021.633586

**Published:** 2021-05-12

**Authors:** Milos Gojkovic, Pedro P. Cunha, Gabriella S. Darmasaputra, Laura Barbieri, Helene Rundqvist, Pedro Veliça, Randall S. Johnson

**Affiliations:** ^1^ Department of Cell and Molecular Biology, Karolinska Institute, Stockholm, Sweden; ^2^ Department of Laboratory Medicine, Karolinska Institute, Stockholm, Sweden; ^3^ Department of Physiology Development and Neuroscience, University of Cambridge, Cambridge, United Kingdom

**Keywords:** Nitric oxide, hypoxia, immunosuppression, HIF, myeloid cells, von Hippel-Lindau., Prolyl hydroxylase inhibitors

## Abstract

Myeloid cell interactions with cells of the adaptive immune system are an essential aspect of immunity. A key aspect of that interrelationship is its modulation by the microenvironment. Oxygen is known to influence myelosuppression of T cell activation in part *via* the Hypoxia inducible (HIF) transcription factors. A number of drugs that act on the HIF pathway are currently in clinical use and it is important to evaluate how they act on immune cell function as part of a better understanding of how they will influence patient outcomes. We show here that increased activation of the HIF pathway, either through deletion of the negative regulator of HIF, the von Hippel-Lindau (VHL) gene, in myeloid cells, or through pharmacological inhibitors of VHL-mediated degradation of HIF, potently suppresses T cell proliferation in myeloid cell/T cell culture. These data demonstrate that both pharmacological and genetic activation of HIF in myeloid cells can suppress adaptive cell immune response.

## Introduction

Oxygen availability is a critical factor in immune response (1). Each cell in the immune system has to carry out its functions in an environment marked by a specific level of oxygenation (2). As immune cells move through tissues, oxygen availability can rapidly change, and these cells thus need to be able to function in high or low oxygen environments (2).

The discovery of the HIF transcription factor, and the finding that HIF and hypoxic response are fundamental elements of immune cell function, has led to numerous important findings concerning the relationships between HIF and immunity (1, 3). These findings have primarily focused on individual cell types and their responses to hypoxia: studies have, for example, found important roles for HIF response in the innate immune system, including macrophages, neutrophils, and dendritic cells, as well as in adaptive immunity in B cells and CD4^+^ and CD8^+^ T cells (3–6).

With this understanding of how oxygenation and hypoxic response influences individual cells it becomes important to understand how these affect cell-cell interactions during immune response. One key finding in this regard was the demonstration that hypoxia can trigger myeloid suppression of T cell proliferation, and that this suppression requires activation of HIF-1α in myeloid cells (7).

Solid tumors often contain immunosuppressive microenvironments (8) characterized by cytokines (including IL10, IL6, galectin-1 and TGF-b) that are upregulated by hypoxia and HIF signaling (9, 10). PD-L1, a protein that blocks cytotoxic T cell function, shows increased expression in both tumor cells and myeloid cells and its expression is in part regulated by HIF-dependent pathways (10). Loss of myeloid HIF-1α results in impaired myeloid cell infiltration and inflammation (3, 11, 12) and decreased tumor progression in a spontaneous mammalian breast cancer model (7). Loss of myeloid HIF-2α has been linked to decreased tumor inflammation and progression in murine models of breast and gastric cancer (13, 14), while tumor-associated myeloid cells can also contribute to immune suppression by production of nitric oxide (NO), acting through the HIF-1α-NOS2 pathway (4, 7).

In this study, we examine the role of deregulated hypoxic response to determine in detail how key HIF-regulated myelosuppressive actors influence cytotoxic T cell division, and how pharmacological manipulation of this pathway might influence immune response.

## Material and Methods

### Animals

VHL^fl/fl^ LysM^Cre+^, HIF-1α^fl/fl^ LysM^Cre+^, HIF-2α^fl/fl^ LysM^Cre+^, Arg-1^fl/fl^ LysM^Cre+^,^+^, Arg-2^fl/fl^ LysM^Cre+^, Arg-2^fl/fl^ LysM^Cre+^ were generated by crossing transgenic mice homozygous for loxP-flanked genes of interest (fl/fl) with mice expressing Cre recombinase driven by the lysozyme M promoter (LysM^Cre+^) (15). Transgenic mice heterozygous for Cre recombinase and homozygous for loxP-flanked genes of interest were the conditional knockouts, and compared to littermates carrying only the loxP-flanked gene of interest as wild type controls. Spleens and lymph nodes were harvested from OT-I mice (003831, The Jackson Laboratory) for isolation of ovalbumin-specific OT-I CD8^+^ T cells. All *in vivo* experiments were approved by the Swedish ethical approval board (Stockholm north, N101/16) and were performed on mice aged between 8-16 weeks. Swedish national guidelines were conformed to in all animal housing and care.

### Cell Lines

All tumor cell lines were cultured in DMEM (11995065, Gibco) supplemented with 10% FBS (10270106, Gibco) and 1% penicillin/streptomycin (10378016, Gibco).

B16-F10-OVA were generated through co-transfection of the transposon vector pT2 containing codon-optimized genes for chicken ovalbumin (OVA; P01012.2), eGFP (ABG78037.1), neomycin phosphotransferase (NeoR; BAD00047.1) and the vector encoding transposase SB11.

OVA, eGFP and NeoR were expressed as a polycistronic peptide interspersed with P2A and furin cleavage sites and synthesized by Gene Art (Thermo Fisher). This was then cloned under the promoter SV40 in the transposon vector pT27BH (gift from Perry Hackett, Addgene plasmid #26556). Plasmid containg the sleeping beauty transposase (pCMV-SB11, Addgene plasmid #26552) was a gift from Perry Hackett.

Transfected cells were cultured with 400 mg/mL G418 (10131027, Gibco) three days post transfection in order to select for transfected cells. Transfection success was confirmed through flowcytometry analysis of eGFP fluorescence. Clonal B16-F10-OVA cell line was then produced through limiting dilution.

### Antigen Presenting Assay

Bone marrow-derived myeloid cells (BMDM) were generated by isolation of bone marrow cells from femur and tibia (16), and cultured for 7 days in DMEM (11995065, Gibco) supplemented with 10% FBS (10270106, Gibco), 1% penicillin/streptomycin (10378016, Gibco), 10ng/mL M-CSF and GM-CSF (416-ML-050/CF and 415-ML-050/CF, R&D Systems). 7 days post isolation, 100ng/mL LPS (L2630, Sigma-Aldrich) was added to culture for 24 hours. For the antigen presenting assay, BMDMs were harvested and incubated with for 1 hour with 100 ng/mL chicken albumin (OVA) derived peptide (SIINFEKL) (S7951, Sigma Aldrich), followed by washing three times with PBS (14190-144, Gibco) and culturing in RPMI (11875085, Gibco) supplemented with 10% FBS, 1% penicillin/streptomycin and 55μM 2-Mercaptoethanol (21985023, Gibco) in 96 well round bottom plates (50 000 cells/well). Surface expression of coactivators and antigen presentation on BMDMs was confirmed by flow cytometry. CD8^+^ OT-I T cells were isolated from gender matched OT-I spleens and lymph nodes by magnetic bead separation (130-104-075, Miltenyi) and stained with CellTrace Violet Cell dye (C34557, Invitrogen), for cell division tracking. The antigen presenting assay was carried out by adding 200 000 T cells to SIINFEKL-loaded BMDM in a 96 well plate. The co-culture was either preformed at atmospheric oxygen conditions (21% O_2_) or at hypoxic conditions (5% or 1% O_2_) for three days in a hypoxia workstation (INVIVO_2_ 400, Ruskinn). Cell division and phenotype was assessed by flow cytometry. CD8^+^ OT-I T cells cultured in media with 100 ng/mL SIINFEKL were used as positive control.

### Flow Cytometry

Cell cultures were harvested and stained with Near-IR Dead Cell Stain Kit (L34975, Thermo Fisher) for dead cell exclusion, followed by surface staining with fluorochrome labeled antibodies (**Table 1**). BD Cytofix/Cytoperm kit (554714, BD Biosciences) was used for cytoplasmic staining of Granzyme B and CTLA-4.

**Table 1:** Antibodies and stains used for different analyses performed with flow cytometry.

Stain name	Antigen	Fluorochrome	Dilution	Clone
Antigen Presenting Assay	CTV	eFluor 450		
	CD8	Brilliant Violet 510	400	53-6,7
	Live/dead	APC-Cy7	500	
	CTLA-4	APC	200	UC10-4F10-11
	GzmB	PE	200	GB12
	CD44	PE-Cy7	400	IM7
BMDM 1	Live/dead	APC-Cy7	500	
	Fc block		50	93
	CD80	Pacific Blue	200	16-10A1
	CD86	Alexa Fluor 488	200	GL-1
	H2KB-OVA	PE	200	25-D1.16
	CD11c	PE-Cy7	125	HL3
	H2KB	Alexa Fluor 647	200	AF6-88.5
BMDM 2	Live/dead	APC-Cy7	500	
	Fc block		50	93
	PD-L1	PE	100	MIH5
	CD11c	PE-Cy7	125	HL3
	MHC Class II	Alexa Fluor 647	1600	M5/114.15.2
Human Monocyte	Live/dead	APC-Cy7	500	
	Fc block		50	
	CD16	Horizon V450	200	3G8
	CD45	FITC	200	HI30
	CD11b	PE	200	M1/70
	CD25	Alexa Fluor 647	200	M-A251
Human *Ex Vivo* Lymphoid	CTV	eFluor 450		
	Live/dead	APC-Cy7	500	
	Fc block		50	
	CD8	BV510	200	SK1
	CD45RA	APC	100	HI100
	TCR α/β	PerCP/Cy5.5	200	IP26
	CD45RO	FITC	100	UCHL1
	GzmB	PE	200	GB12

Viability staining was performed using or Near-IR Dead Cell Stain Kit (L34975, Thermo Fisher).

### NOC-18 Treatment

100 000 CD8^+^ T cells were activated in 96-well round bottom plates with CD3/CD28 Dynabeads (11453D, Thermo Fisher) in 200uL RPMI supplemented with 10% FBS, 1% penicillin/streptomycin and 2-Mercaptoethanol and 10U/mL IL-2 in the presence of increasing NOC-18 (ALX-430-014-5005, Enzo) concentrations (12.5, 25, 100, 200 and 400 μM). For cell division tracking, cells were loaded with CTV. 72 hours after activation T cells were analyzed by flow cytometry.

### Tumor Assays

Transgenic mice were injected with 2x10^5^ B16-F10-OVA intravenously by the tail vein. Lungs were perfused with PBS through the left heart ventricle and collected from euthanized mice 21 days post injection of tumor cells. The number of metastatic foci was determined by counting black nodules macroscopically and sized by scoring system.

### Antigen Presenting Assay with FG4592 and DMOG

For optimization, BMDMs were treated with 100, 50, 25, 12.5, 0 μM FG4592 (HIF prolyl-hydroxylase inhibitor) or hypoxia for 24 hours before collected for transcriptional analysis of iNOS expression using Qiagen RNeasy Kit (cat nr: 74106). cDNA was produced with iScript cDNA Synthesis Kit (1708890, Bio Rad) followed by realtime analysis using KiCqStart SYBR Green predesigned primers (KSPQ12012, Sigma-Aldrich) for Nos2 (GeneID: 18126) and Hprt (GeneID: 15452). The optimal dose of FG4592 was selected by choosing the concentration of FG4592 that induces the same level of Nos2 expression as hypoxia. The antigen presenting assay was performed as described above with either 12,5 μM FG4592 (15294, Cayman Chemical), 12,5 μM DMOG (71210 Cayman Chemical) or Dimethyl sulfoxide (D8418, Sigma-Aldrich) as a solvent control.

### Myeloid Suppression Assay

BMDMs and CD8+ T cells were acquired and stained (as described above) from transgenic and wildtype mice respectively. CTV stained CD8+ T cells were then activated with Dynabeads Mouse T-Activator CD3/CD28 (ThermoFisher, Sweden) for 24 hours. Prior to the co-culture with myeloid cells, Dynabeads were removed from CD8^+^ T cell culture by magnetic separation. Myeloid suppression assay was performed by co-culture of 50 000 LPS activated BMDM with 200 000 activated T cells for 72 hours in a round bottom 96 well plate.

Myeloid suppression assays with human primary cells were performed by isolation of peripheral blood monocytes (PBMC) using Ficoll-Paque PLUS (17-1440-02, GE Healthcare) from buffy coats provided from Karolinska University Hospital (Stockholm, Sweden). CD8^+^ T cells and monocytes were isolated by microbead separation using positive selection for CD8^+^ T cells (130-045-201, Miltenyi) followed by negative selection for pan-monocytes (130-096-537, Miltenyi). Prior to co-culture of monocytes and CD8^+^ T cells, 100 ng/mL LPS was added to the monocyte culture, while CD8^+^ T cells were activated using plate bound Ultra-LEAF Purified anti-human CD3 antibody (clone OKT3, Biolegend). 24 hours post activation monocytes and CD8^+^ T cells were washed once with complete RPMI cell media and co-cultured for 72 hours in a round bottom 96 well plate (50 000 monocytes and 200 000 CD8^+^ T cells per well), followed by analysis with flow cytometry. As positive controls activated CD8^+^ T cells were cultured with plate bound anti-human CD3 antibody with similar treatments and conditions as co-cultured samples. All cell cultures were performed in RPMI supplemented with 10% FBS, 1% penicillin/streptomycin and 55μM 2-Mercaptoethanol.

## Results

In order to better understand oxygen-mediated changes in myeloid/lymphoid interactions, we first sought to determine whether increased expression of HIF transcription factors in myeloid cells can drive immunosuppression during antigen-dependent CD8^+^ T cell activation. We utilized a co-culture system in which myeloid cells present a specific antigen (the ovalbumin-derived OVA peptide) to transgenic CD8+ T cells bearing the OT-I T cell receptor specific for that peptide (OT-I T cells).

We undertook co-culture of bone marrow-derived myeloid cells (BMDMs) which were LPS-activated and loaded with the OVA peptide SIINFEKL. The myeloid cells present antigen through H-2Kb to naïve CD8^+^ T cells from transgenic mice expressing the OT-1 transgene, specific for the SIINFEKL peptide (this experimental model is outlined in **Supplementary Figures 1A–C**). As a positive control, OT-I CD8^+^ T cells were cultured with SIINFEKL peptide, but without BMDMs. As shown in representative histograms (**Supplementary Figure 1D**), there were no significant oxygen-dependent effects seen on CD8^+^ T cell division or activation (**Supplementary Figure 1D**) detected after three days in this experimental assay.

**Figure 1:**
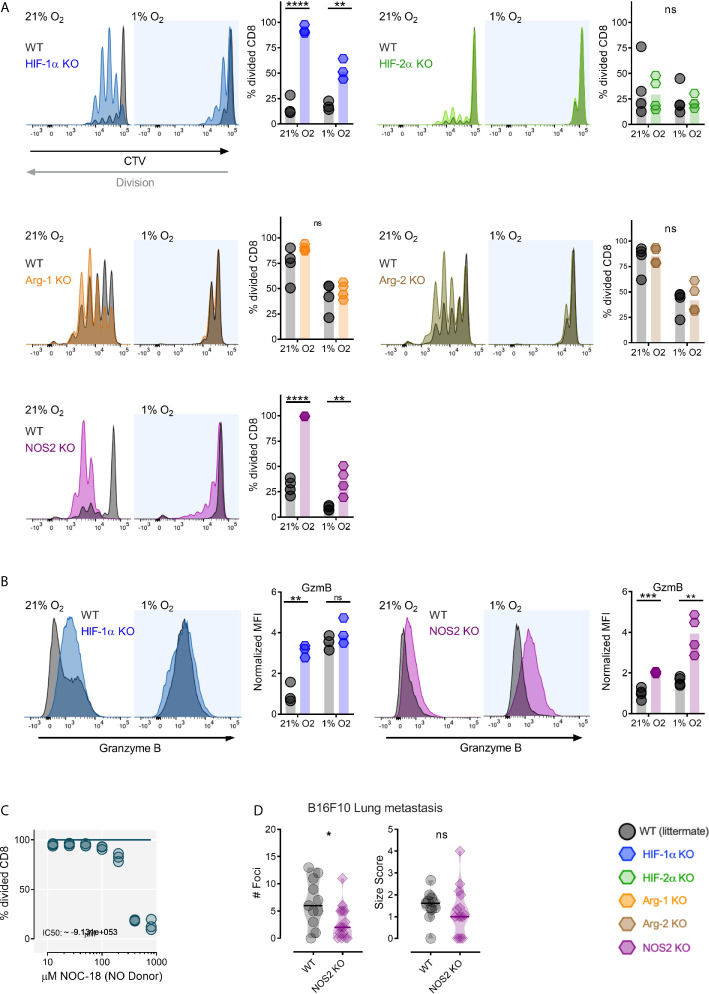
Myeloid HIF-1α inhibits antigen induced CD8^+^ T-cell division through NOS2. **(A)** Antigen presenting assay showing representative CTV histograms and division fraction of CD8^+^ T-Cells co-cultured with HIF-1α ^fl/fl^ (WT) and HIF-1α ^fl/fl^ LysM^Cre+^ (HIF-1α KO), HIF-2α ^fl/fl^ (WT) and HIF-2α ^fl/fl^ LysM^Cre+^ (HIF-2α KO), Arg-1^fl/fl^ (WT) and Arg-1^fl/fl^ LysM^Cre+^ (Arg-1 KO), Arg-2^fl/fl^ (WT) and Arg-2^fl/fl^ LysM^Cre+^ (Arg-2 KO), NOS2^fl/fl^ (WT) and NOS2^fl/fl^ LysM^Cre+^ (NOS2 KO) BMDMs in 21% or 1% oxygen. **(B)** Expression of Granzyme B as representative histograms and normalized MFI of CD8^+^ T-Cells activated by antigen presenting HIF-1α ^fl/fl^ *vs* HIF-1α ^fl/fl^ LysM^Cre+^ and NOS2^fl/fl^ *vs* NOS2^fl/fl^ LysM^Cre+^ BMDMs in 21% or 1% oxygen. **(C)** CD8^+^ T-cells activated for 72 hours in 5% O_2_ and cultured for 2 days with increasing concentration of nitric oxide donor (NO) NOC-18. n=3 donors **(D)** 10-13 week old mice were injected intra venously with 0.2x10^6^ B16-F10-OVA tumor cells. The mice were sacrificed 21 days post injection for collection of lungs. Number of foci and size score NOS2^fl/fl^ *vs* NOS2^fl/fl^ LysM^Cre+^ mice. Data presented as scatter dot plots or violin plots, *P < 0.05, **P < 0.01, ***P < 0.001, ****P < 0.0001; ns, not significant. Statistical analysis was performed with unpaired T test, n= 3-4 bone marrow donors **(A, B)** and n= 11-15 mice per group **(D)**.

Previous work from our group has found that T cell proliferation following TCR triggering is suppressed in hypoxia during co-culture with wild type myeloid cells, and found this suppression to be dependent on an intact myeloid cell HIF-1α gene (7). To further examine this, the genetic components of hypoxia-driven suppression of CD8^+^ T cell division by myeloid cells were determined. As shown in **Figure 1A**, loss of HIF-1α in myeloid cells increased cell division in co-cultured CD8^+^ OT-I expressing T cells under both high and low oxygen conditions following activation by antigen presenting myeloid cells. Conversely, loss of HIF-2α (**Figure 1A**) in myeloid cells had no significant effect on co-cultured antigen activated T cells during either high or low oxygen culture. This indicates that in this experimental system, the key transcriptional effector of myeloid immunosuppression caused by hypoxia is HIF-1α.

We next examined a number of HIF target genes that regulate nitric oxide that are known to induce myeloid immunosuppression of T cell responses (4); these genes can either act to suppress nitric oxide production (Arg1 and Arg2) or induce it (NOS2). Arg1 and Arg2 have been shown to be HIF-2α target genes in myeloid cells, whereas NOS2 is primarily regulated by HIF-1α in these cells (4, 17). Arg1 deletion in myeloid cells (using Arg1^fl/fl^ and Arg1^fl/fl^ LysM^Cre+^strain mice) results in an insignificant increase in T cell division in high oxygen, with little significant change in division in low oxygen (**Figure 1A**). However, Arg2 deletion (using Arg2^fl/fl^ and Arg2^fl/fl^ LysM^Cre+^strain mice) has little effect on T cell division in co-culture (**Figure 1A**). This is consistent with the lower levels of Arg2 expression seen in myeloid cells relative to Arg1 expression. Interestingly, loss of myeloid cell NOS2 (using NOS2^fl/fl^ and NOS2^fl/fl^ LysM^Cre+^strain mice) allows increased T cell antigen presentation-induced division in both high and low oxygen conditions relative to co-culture with wild type myeloid cells (**Figure 1A**).

Loss of myeloid cell HIF-1α caused a post-activation increase in expression of the activation marker Granzyme B on CD8^+^ T cells during high oxygen co-culture; whereas loss of NOS2 in myeloid cells increased Granzyme B expression under both low and high oxygen co-culture conditions (**Figure 1B**). As can be seen in **Figure 1C**, increasing concentrations of an NO donor molecule, NOC-18, effectively suppresses division of activated T cells; this occurs in a dose-dependent manner.

As myeloid cells are known to have an important impact on establishment of metastatic tumors (18), mice with a loss of function of the NOS2 gene in myeloid cells (using NOS2^fl/fl^ and NOS2^fl/fl^ LysM^Cre+^strain mice) were injected intravenously with OVA expressing B16-F10 cells, a metastatic melanoma cell line. As shown here, mice lacking myeloid NOS2 had decreased numbers of metastatic foci (**Figure 1D**). This indicates that in this model, myeloid NOS2 expression acts to facilitate metastatic tumor initiation, and may affect overall growth of these tumors, although there was no significant effect of this mutation on metastatic tumor size in these experiments.

**Figure 2:**
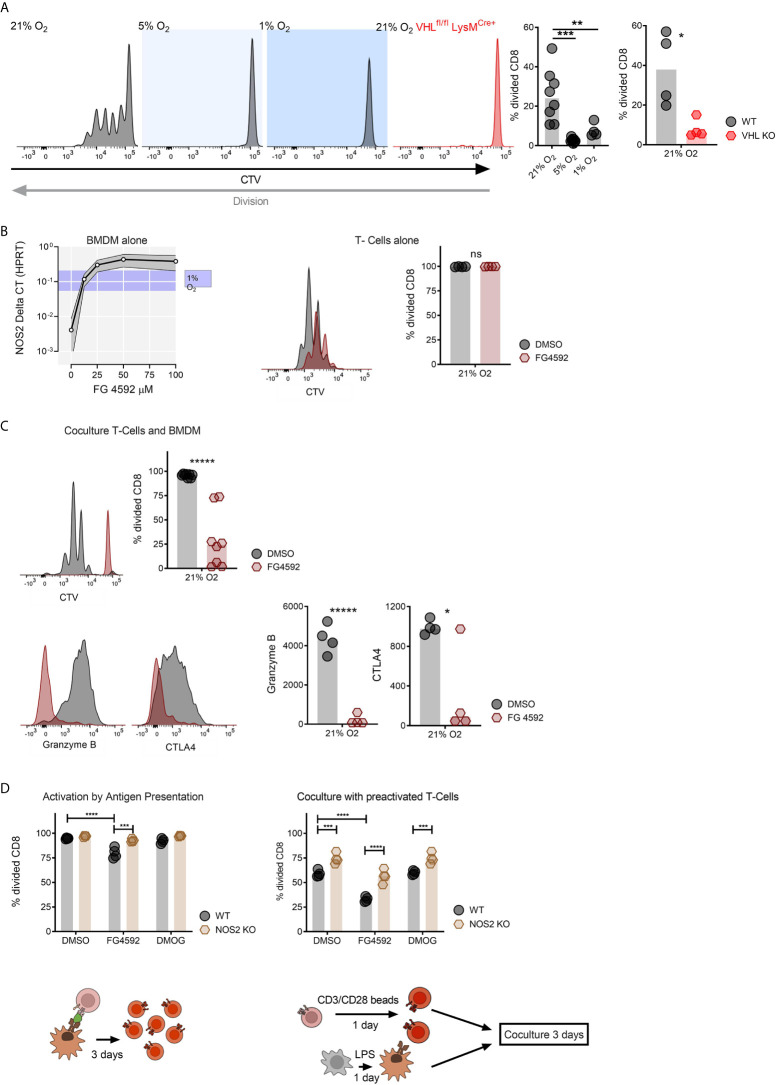
Genetically and compound induced myeloid hypoxia blocks CD8^+^ T-cell division *in vitro*. **(A)** Representative flow cytometry histograms of CTV stained CD8^+^ T-cells co-cultured with OVA presenting BMDMs in 21%, 5%, 1% oxygen and with BMDMs without Von Hippel-Lindau (VHL^fl/fl^ LysM^Cre+^) in 21% oxygen, division fraction of CD8^+^ T-cells co-cultured with antigen presenting wildtype BMDMs in different oxygen levels and division fraction of CD8^+^ T-cells co-cultured with VHL^fl/fl^ (WT) *vs* VHL^fl/fl^ LysM^Cre+^ (VHL KO) BMDMs in 21% oxygen. **(B)** NOS2 mRNA expression in BMDMs cultured with increasing concentration of FG-4592 (HIF prolyl-hydroxylase inhibitor) or hypoxia (with ranging values demonstrated as blue shade on y axis) and CD8^+^ T-cells in a single culture with DMSO or 12.5 µM FG-4592. **(C)** Antigen presenting assay; BMDM and CD8^+^ T-cell co-culture treated either with FG-4592 compared to DMSO controls and expression of Granzyme B in CD8^+^ T-cells in co-culture with BMDMs. **(D)** Antigen presentation assay performed with NOS2^fl/fl^ or NOS2^fl/fl^ LysM^Cre+^ BMDMs in 21% treated with 12.5 µM FG-4592 or DMOG and co-culture of pre-activated BMDM from NOS2^fl/fl^ or NOS2^fl/fl^ LysM^Cre+^ mice and pre-activated CD8^+^ T-cells from wildtype mice. Co-cultures were performed in media containing DMSO, 12.5 µM FG-4592 or DMOG. Data presented as scatter dot plots, *P < 0.05, **P < 0.01, ***P < 0.001, ****P < 0.0001, *****P < 0.00001; ns, not significant. Statistical analysis was performed with unpaired T test, n= 4-8 bone marrow donors/group.

To determine whether increased amounts of HIF expression from myeloid cells inhibits T cell division under high oxygen co-culture, we employed the antigen presentation conditions described above with CD8^+^ T cells and myeloid cells with a null mutation in the VHL gene (using VHL^fl/fl^ and VHL^fl/fl^ LysM^Cre+^strain mice). These mutant mice have increased levels of HIF expression under high oxygen conditions due to diminished oxygen-mediated HIFα degradation. As can be seen in **Figure 2A**, while wild type myeloid cells suppress T cell division after activation in an oxygen-dependent manner during co-culture, VHL mutant myeloid cells completely suppress T cell division, even under high oxygen conditions.

A number of inhibitors of the prolyl hydroxylase enzymes are currently either in clinical use or under evaluation, primarily for the treatment of anemia (19). These inhibitors act to block hydroxylation of two of the proline residues in the HIF-alpha proteins, causing them to become resistant to oxygen-mediated degradation by VHL complexes. As a functional read-out of this effect, we show in **Figure 2B** that stabilization of HIF by the prolyl hydroxylase inhibitor FG4592 (20, 21) gives rise to a dose-dependent increase in HIF-dependent NOS2 mRNA transcription in primary myeloid cells. In **Figure 2B** we also show that activated CD8^+^ T cells cultured alone or treated with 12.5 micromolar levels of the prolyl hydroxylase inhibitor continue to proliferate normally. However, as can be seen in **Figure 2C**, in a co-culture system, the prolyl hydroxylase inhibitor has a strongly inhibitory effect on T cell division. This extends to inhibition of the expression of a number of markers of T cell activation, including Granzyme B, and CTLA4 (**Figure 2C**).

In order to ascertain whether this myeloid suppression of CD8^+^ T cell division is NO-dependent, we performed the antigen presenting co-culture assay described above with NOS2 deleted myeloid cells, in combination with one or the other of the prolyl hydroxylase inhibitors FG4592 and DMOG (**Figure 2D**) (21, 22) We have previously shown that the NOS2 gene is the predominant source for NO in activated murine myeloid cells (4, 7). As shown in **Figure 2D**, suppression of myeloid NO production *via* deletion of the myeloid NOS2 gene significantly reduced the myelosuppression caused by these two prolyl hydroxylase inhibitors in co-culture (**Figure 2D**). This indicates that a key aspect of the myelosuppression caused by pharmacological activation of HIF is NO production by myeloid cells.

**Figure 3:**
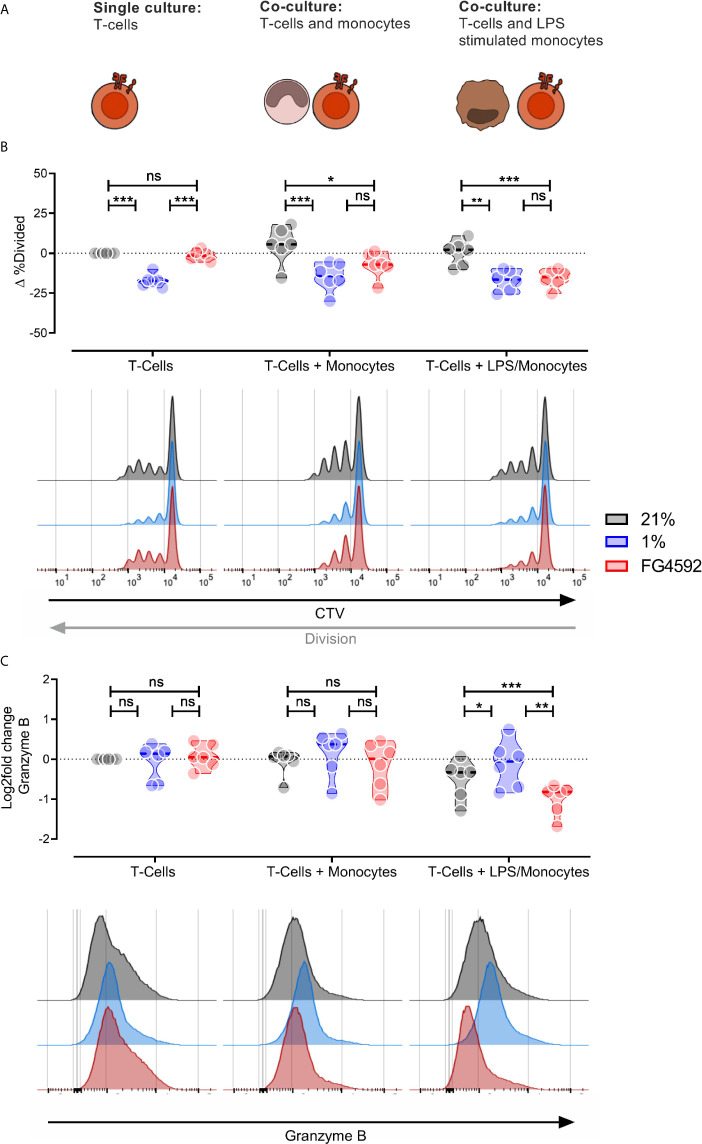
Human monocyte compound induced HIF response blocks human T-cell division *in vitro*. **(A)** Illustration of the experimental layout. Human monocytes and CD8^+^ T-cells were isolated from peripheral blood mononuclear cells and activated with 100 ng/mL LPS and plate bound anti-CD3 antibodies (respectively) for 24 hours. Activation was followed by culturing activated CD8^+^ T-cells alone or co-culturing CD8^+^ T-cells with either unstimulated or LPS stimulated monocytes. **(B)** Percentage divided CD8^+^ T-Cells after three days in three different culturing conditions and treatments; DMSO, 1% O_2_ or 12.5 µM. All samples presented as the difference to untreated single cultured donor-matched CD8^+^ T-cells. **(C)** Expression of Granzyme B in CD8^+^ T-cells treated as in **(B)**. Data is log_2_ fold change MFI relative to untreated single cultured donor-matched CD8^+^ T-cells. Data presented as violin plots or representative histograms, *P < 0.05, **P < 0.01, ***P < 0.001; ns, not significant. Statistical analysis was performed with Two-way RM ANOVA and Tukey’s multiple comparisons test, n = 6 blood donors/group.

As these experiments were all carried out in murine systems, in order to investigate whether these findings are relevant to human cells, we carried out a myeloid suppression assay using human donor peripheral blood monocyte-derived monocytes stimulated *ex vivo* with LPS (**Supplementary Figure 2A**) and CD8^+^ T cells (**Supplementary Figure 2B**), either in hypoxia, or with FG4592 (**Figure 3A**). We found that, as with murine CD8^+^ T cells, FG4592 does not alter CD8^+^ T cell division in a monoculture of cytotoxic T cells; however, in co-culture with monocytes, FG4592 causes significant suppression of CD8^+^ T cell division (**Figure 3B**). Co-cultures of LPS-stimulated monocytes and CD8^+^ T cells with FG4592 showed suppression of CD8^+^ T cell expression of the T cell effector molecules Granzyme B (**Figure 3C**) and CD45RO (**Supplementary Figure 2C**), while leaving expression of the marker CD45RA unaffected (**Supplementary Figure 2D**). Overall, this data confirms that FG4592 enhances myeloid cell suppression of CD8^+^ T cell division and activation in both murine and human primary cell cultures.

## Discussion

We show here that even intermediate levels of hypoxia can act to trigger myelosuppressive properties in co-culture of myeloid cells with CD8^+^ T cells. Follow up experiments demonstrated that myeloid inhibition of T cell proliferation was dependent on NO production, specifically through the action of the HIF-1α transcription factor. LPS-stimulated myeloid cells will produce significant amounts of NO even in 21% O_2_ (23), however, lowering oxygen levels increases nitric oxide production and therefore increases myelosuppression, explaining the observation that even at high oxygen levels, deletion of myeloid HIF-1α or NOS2 allows increased T cell activation and expansion.

Activation of cytotoxic T cells in the presence of the nitric oxide donor NOC-18 suggests that inhibition by NO is dose dependent, reinforcing the hypothesis that the effect of the highly labile NO molecule is strongest when myeloid cells and T cells are in close proximity to one another, such as when an immunological synapse is formed. The reduced numbers of metastases seen here in mice lacking myeloid NOS2 suggest that myeloid NO may contribute to early stages of tumor establishment, particularly as the size of metastatic lesions was not significantly different between wild type and NOS2 mutant mice.

Genetic and pharmacological induction of myeloid HIF-1α (either through deletion of VHL or treatment with FG4592) demonstrated a strong connection between HIF-1α-driven NO production and the ability of myeloid cells to suppress cytotoxic T cell division and activation. As shown in **Figure 2D**, this myeloid suppression is independent of whether T cells are activated independently first, or activated directly in the presence of myeloid cells.

It should be noted that there is extensive evidence for interaction of the NF-kB pathway and HIF and hypoxia (24). An important element for future investigation of the mechanisms of hydroxylase inhibition and the interaction between the innate and adaptive immune system will necessarily need to take this into account. Future studies should help to determine more exactly when hypoxia and HIF activation effect T cell proliferation following antigen presentation and recognition. The timing of these events relative to oxygenation flux will likely be in part dependent on when NF-kB function is itself triggered or suppressed.

Inhibition of PHD activity has been shown to have a potent therapeutic value in models of inflammatory diseases such as colitis (25). Studies have also shown that genetic deletion of PHD-1 in murine models of dextran sulfate sodium (DSS) -induced colitis protected against disease (26). Furthermore, various pharmacological compounds that influence the HIF pathway have demonstrated a protective role in murine models of colitis, and in suppression of disease associated inflammation (27–30). Thus, one possible interpretation of our data is that some of these findings cited above of an anti-inflammatory role for HIF may be due to a HIF-regulated myeloid cell suppression of T cell-induced or -dependent tissue damage.

FG4592 has recently been approved for treatment of anemia in patients suffering from chronic kidney diseases (31). Our data present an interesting and potentially important concern regarding the effects of this and similar drugs on the immune system, and in particular their potential role in modulating myelosuppression.

## Data Availability Statement

All data involved in this article is available on request from the corresponding author.

## Ethics Statement

Animal handling and health monitoring was preformed according to national guidelines for animal care and research. All experiments in this study have been reviewed and approved by the Stockholm north ethical committee (N101/16).

## Authors contributions

MG, PPC, PV, HR and RJ designed and interpreted the experiments in this study. MG, PPC, GD, LB and PV were involved in data collection. MG, RJ, PV and HR wrote the manuscript. All authors reviewed and approved the final manuscript.

## Funding

This study was funded by the Swedish Research Council (Vetenskapsrådet 2019-01485), the Swedish Cancer Society (Cancerfonden CAN2018/808), the Swedish Children’s Cancer Society (Barncancerfonden PR2020-0075) the Knut and Alice Wallenberg Foundation Scholars Award, and the Wellcome Trust, UK (Principal Research Fellowship 214283/Z/18/Z). PPC was supported by a Portuguese Foundation for Science and Technology (FCT) scholarship (SFRH/BD/115612/2016).

## Conflict of Interest

The authors declare that the research was conducted in the absence of any commercial or financial relationships that could be construed as a potential conflict of interest.​​

## Supplementary Material

Supplementary Figure 1 The antigen presenting assay. **(A)** Illustration of the *in vitro* antigen presenting assay. Bone marrow derived myeloid cells (BMDM) were activated with 100 ng/mL LPS for one day, followed by 1-hour incubation with 100 ng/mL OVA; 257-264 (SIINFEKL). CellTrace Violet Staining (CTV) stained CD8^+^ OT-I T-Cells were co-cultured with BMDMs for 3 days. **(B)** Representative gating strategy used to analyze BMDMs surface markers, and co-activators and antigen presenting surface proteins were upregulated in BMDMs stimulated with LPS. **(C)** Gating strategy to measure OT-I T-Cell division and surface markers. Expression level of surface markers was determined on the whole live CD8^+^ population whereas division level was defined by fraction of divided live CD8^+^ T-cells. **(D)** Representative CTV plots of negative and positive controls for antigen presenting assays with division fraction of untreated CD8^+^ T-cells (negative control) or activated by directly adding 100 ng/mL SIINFEKL to culturing media (positive control) without any presence of bone marrow derived myeloid cells. Bottom row shows representative histograms and normalized median florescence intensity (MFI) of Granzyme B and CD44 expression in activated CD8^+^ T-cells, cultured in 21%, 5% and 1% O_2_. MFI values were normalized to 21% oxygen. Data presented as scatter dot plots, *P< 0.05, **P< 0.01, ***P<0.001, ****P< 0.0001; ns, not significant. Statistical analysis was performed with unpaired T test, n = 8 bone marrow donors/group or 6-3 CD8^+^ OT-I T-Cell donors/group.

Supplementary Figure 2 Human monocyte compound induced hypoxia alters expression of human CD8^+^ T-cell activation markers. **(A)** Representative gating strategy used to analyze monocyte surface markers followed by scatter dot plots and representative histograms of CD25 and CD16 surface expression of monocytes with or without LPS treatments. Statistical analysis was performed with (donor) paired T-test. **(B)** Representative gating strategy used to analyze CD8^+^ T-cell division and expression of different surface markers. **(C)** Expression of CD45RO and **(D)** CD45RA as log2fold change in CD8^+^ T-cells from different conditions and treatments. Data presented as violin plots or representative histograms, *P< 0.05, **P< 0.01, ***P<0.001; ns, not significant. Statistical analysis was performed with Two-way RM ANOVA and Tukey’s multiple comparisons test, n = 6 blood donors/group. All samples were standardized to untreated single cultured donor matched CD8^+^ T-cells.
