# Supplementary figures and images for: Oxygen-Mediated Suppression of CD8+ T Cell Proliferation by Macrophages: Role of Pharmacological Inhibitors of HIF Degradation
Source: Front Immunol. 2021 May 12;12:633586. doi: 10.3389/fimmu.2021.633586 (PMC8153186; doi:10.3389/fimmu.2021.633586)

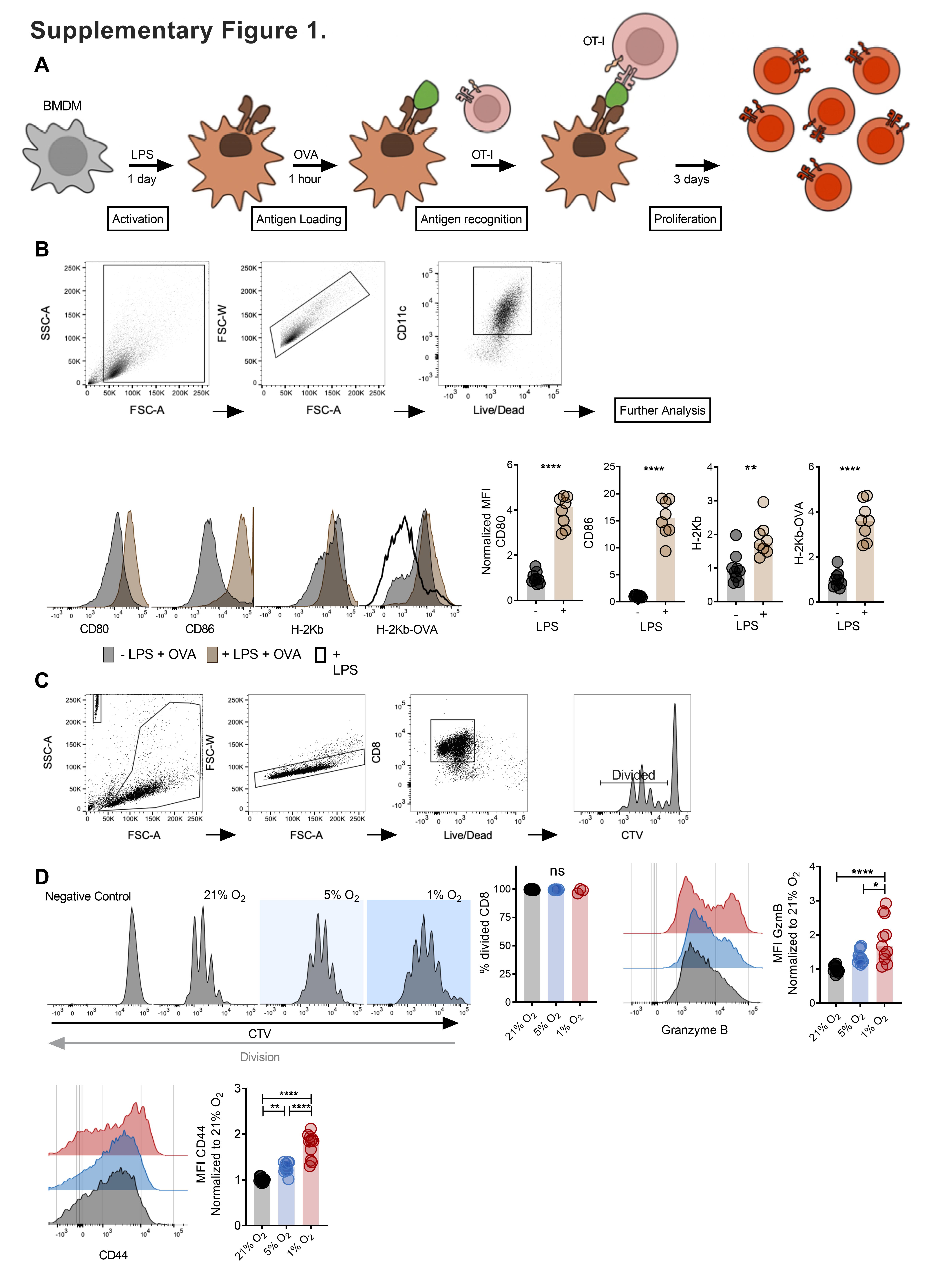

Supplement: Supplementary Figure 1 — The antigen presenting assay. (A) Illustration of the in vitro antigen presenting assay. Bone marrow derived myeloid cells (BMDM) were activated with 100 ng/mL LPS for one day, followed by 1-hour incubation with 100 ng/mL OVA; 257-264 (SIINFEKL). CellTrace Violet Staining (CTV) stained CD8+ OT-I T-Cells were co-cultured with BMDMs for 3 days. (B) Representative gating strategy used to analyze BMDMs surface markers, and co-activators and antigen presenting surface proteins were upregulated in BMDMs stimulated with LPS. (C) Gating strategy to measure OT-I T-Cell division and surface markers. Expression level of surface markers was determined on the whole live CD8+ population whereas division level was defined by fraction of divided live CD8+ T-cells. (D) Representative CTV plots of negative and positive controls for antigen presenting assays with division fraction of untreated CD8+ T-cells (negative control) or activated by directly adding 100 ng/mL SIINFEKL to culturing media (positive control) without any presence of bone marrow derived myeloid cells. Bottom row shows representative histograms and normalized median florescence intensity (MFI) of Granzyme B and CD44 expression in activated CD8+ T-cells, cultured in 21%, 5% and 1% O2. MFI values were normalized to 21% oxygen. Data presented as scatter dot plots, *P< 0.05, **P< 0.01, ***P<0.001, ****P< 0.0001; ns, not significant. Statistical analysis was performed with unpaired T test, n = 8 bone marrow donors/group or 6-3 CD8+ OT-I T-Cell donors/group. [file Image_1.jpeg]

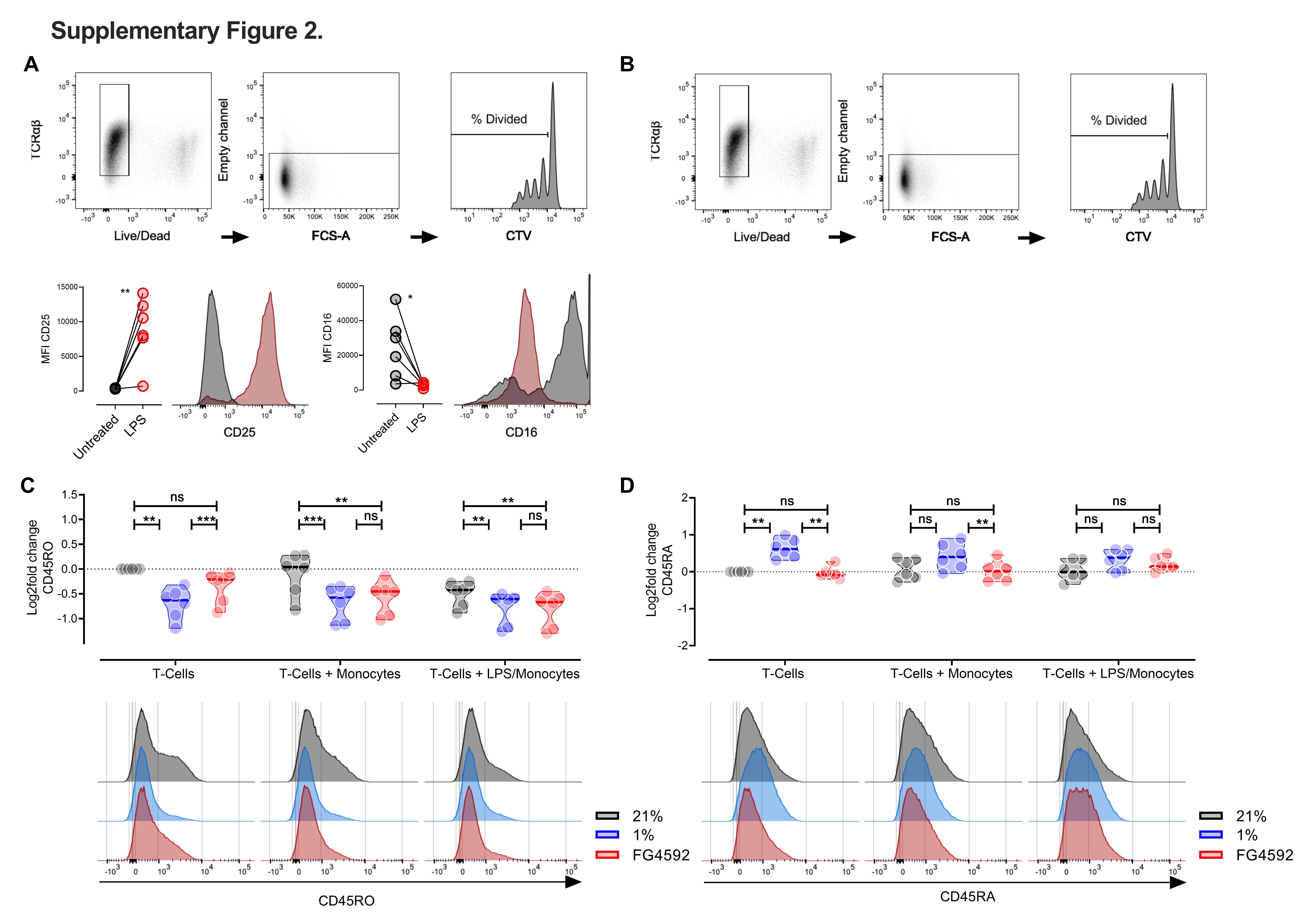

Supplement: Supplementary Figure 2 — Human monocyte compound induced hypoxia alters expression of human CD8+ T-cell activation markers. (A) Representative gating strategy used to analyze monocyte surface markers followed by scatter dot plots and representative histograms of CD25 and CD16 surface expression of monocytes with or without LPS treatments. Statistical analysis was performed with (donor) paired T-test. (B) Representative gating strategy used to analyze CD8+ T-cell division and expression of different surface markers. (C) Expression of CD45RO and (D) CD45RA as log2fold change in CD8+ T-cells from different conditions and treatments. Data presented as violin plots or representative histograms, *P< 0.05, **P< 0.01, ***P<0.001; ns, not significant. Statistical analysis was performed with Two-way RM ANOVA and Tukey’s multiple comparisons test, n = 6 blood donors/group. All samples were standardized to untreated single cultured donor matched CD8+ T-cells. [file Image_2.jpeg]
